# Supplementary material for: Establishing 20S Proteasome Genetic, Translational and Post-Translational Status from Precious Biological and Patient Samples with Top-Down MS
Source: Cells. 2023 Mar 8;12(6):844. doi: 10.3390/cells12060844 (PMC10047880; doi:10.3390/cells12060844)
Supplement: Supplementary file 1 [file cells-12-00844-s001.zip › cells-2111804-supplementary.pdf]

Supplementary Material:

Relative Label-free Quantification of c20S and i20S

1. Establishing response factors for each proteasome subunit

In order to calculate the Relative Ionization Yield (RIY, i.e response factor) of each 20S subunit, we first produced pure c20S and i20S proteasome complexes, using dedicated HEK EBNA cell lines [2,5,8]. As expected, we could only detect the β1/β2/β5 and β1i/β2i/β5i catalytic subunits in the c20S and i20S samples, respectively (**Figure S8A-B**).

The abundances of each subunit were then summed up over the different MS scans and divided by the average abundance of all the non-catalytic subunits, since they are present in all 20S subtypes (**Figure S9**), in order to obtain their RIY. It is important to note here that we assume that each α and β subunit are stoichiometric in these samples, including the bait, α2. This assumption seems reasonable, since a Size Exclusion Chromatography (SEC) was achieved after the immunopurification step, in order to purify fully assembled 20S complexes. Nonetheless, it appears quite clearly that some subunits, such as α2, α5 and β4 have a higher propensity to capture protons in the electrospray, compared to others, such as β1, β2 or β1i. Overall, the RIYs range from 0.31 ± 0.14 (β2i) up to 1.6 ± 0.4 (α5) (**Figure S9**). Importantly, β5, β5i and β1i are the catalytic subunits that show the best ionization yields, close to the average of the other subunits. For that reason, and because β5i is present in all immuno-containing 20S subtypes, including i20S intermediates (containing β1/β2/β5i or β1i/β2/β5i) [2,5], we decided to further use the relative abundance of β5i vs. β5 to semi quantify the immuno-containing 20S complexes.

In order to test the robustness of these estimated RIYs, we repeated the same experiment but after buffer exchanging the 20S complexes in 200 mM ammonium acetate pH 7.4 and performing this time MSMS acquisition. The RIYs were similar for 12 subunits out of 17, but we observed a significant decrease for α4, β4, and β5 and increase for β1 and β7 (**Figure S10**). We expect to have similar or even higher variability from one instrument to another and these results prompted us to establish the RIY before the semi-quantification of any real sample.

2. Benchmark on mixes from purified proteasomes

We then mixed the two prepurified proteasome samples in different proportions (c20S/i20S: 10%/90% - 20%/80% - 30%/70% - 50%/50% - 70%/30% - 80%/20% and 90%/10%), acquired the corresponding proteoform maps in triplicate (**Figure S8C**), then calculated the amount of each subunits by (1) normalizing the abundance of each subunit with the average abundance of all the non-catalytic subunits and by (2) first correcting the abundance of each subunit by dividing them with the corresponding RIY and then normalizing them with the average corrected abundance of all the non-catalytic subunits. Since the non-catalytic subunits are the same in c20S and i20S, we expect them to tend to 1. As shown in **Figure S11A-B**, the relative amounts were obviously nearer to 1 when we applied the RIY correction (**Figure S11B**) than without the correction (**Figure S11A**). Similarly, as shown in **Figure S11C-D**, the correction with RIYs allowed a better estimate on the proportions of the catalytic subunits- thereby on the proportions of c20S and i20S. We assessed the quality of this method by comparing the calculated results with the theoretical abundances of each subunit in these nine mixes. As shown in **Figure S11E**, the accuracy and coefficient of variation were improved with the calculation that includes the correction with RIY.

3. Test-case on precious biological samples:

The next step was to assess this relative quantification technique directly from immunopurified 20S from cells and tissues expressing both c20S and i20S. We first looked into the induction of the immuno subunits in Caco2 cells using IFNγ after 3, 7, and 11 days (**Figure S13A-C**). With our method, we were able to see the decrease in standard catalytic subunits and, concurrently, the increase in immuno subunits upon treatment with IFNγ (see **Figure S13B-C**). As shown in **Figure S13A**, we found better accuracy for the relative amount of non-catalytic subunits (theoretically equal to 1) calculated with correcting first the abundances with the established RIY then normalizing them (median of accuracy at 96 %) than the relative amount calculated with just the normalization (median of accuracy at 88 %). However, the standard deviation was similar at 8% (median of coefficient of variation). To validate the results for the catalytic subunits, we used the relative amount calculated from the label-free

quantification using BU-MS, which our team already established before [50]. We found that on these endogenous samples, there were some inconsistencies on β1 and β2i (**Figure S14**), so β5 and β5i were the only subunits taken into account to approximate the relative abundances of c20S and immuno-containing 20S, respectively. The results for Caco2 cells, as shown in **Figure S13B-C**, indicates that TD-MS quantification with the RIY application (i.e. 129% mean accuracy) is more comparable with the results from BU-MS than without considering the RIY (i.e. 48% mean accuracy).

We also tried the same comparison with an intestinal crypt sample from normal tissue of a patient with suspicion of cancer which had proteasome levels of 90% i20S and 10% c20S as relatively quantified with BU-MS. Similarly, as shown in (**Figure S13D**), the results where RIY correction was applied gave a more accurate result with 94% accuracy than with just the direct normalization. Based on four different TD-MS injections, this sample has 90% ± 9% i20S and 10% ± 4% c20S. Finally, we applied the same approach on BLCLs from PRAAS patients and found that our TD-MS quantification was in better agreement with the BU-MS quantification after correction with the RIYs (**Figure S13E-F**).

Supplementary Figures:

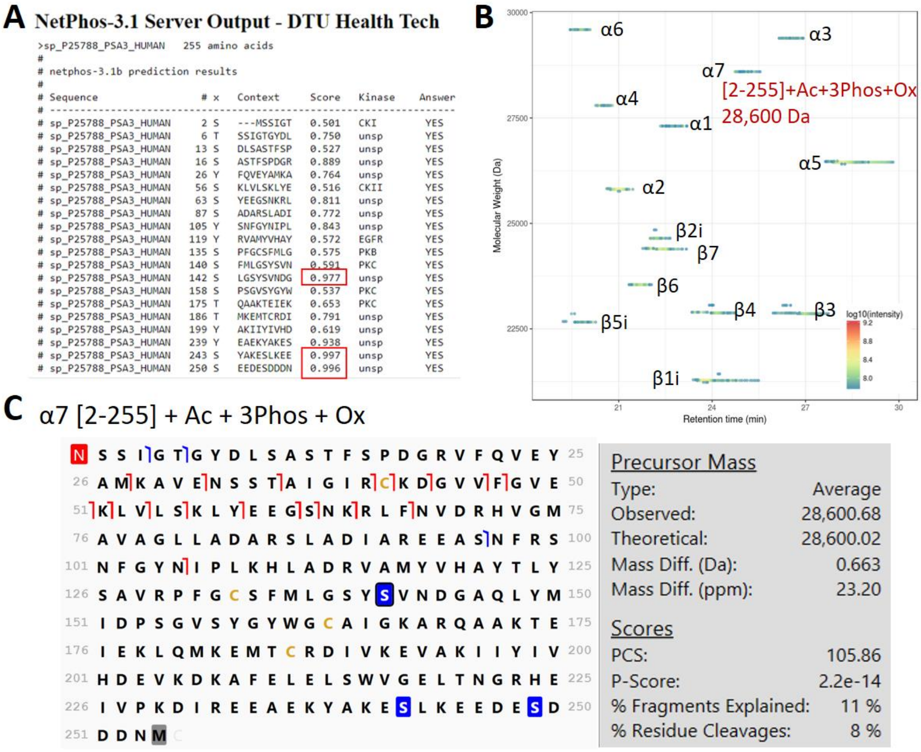

Figure S1. TD-MS identification of proteoforms in human immunoproteasome (i20S) purified from HEK-EBNA cells. (**A**) Prediction of phosphosites (score > 0.5) on *α7* (*PSMA3*) determined by the NetPhos3.1 Server. (**B**) Proteoform map using VisioProtMS showing the i20S subunits showing the triply-phosphorylated form at 28,600 Da as the main proteoform of *α7* (*PSMA3*). Figures in brackets indicate the first and last amino acids in the sequence of the corresponding detected protein. Ac = acetylation, Ox = oxidation, Phos = phosphorylation; and (**C**) its MSMS top-down sequencing using Proteome Discoverer. Acetylation, oxidation and phosphorylation sites are highlighted in red, gray, and blue, respectively. Red and blue lines correspond to c-/z- and b-/y- ions, respectively.

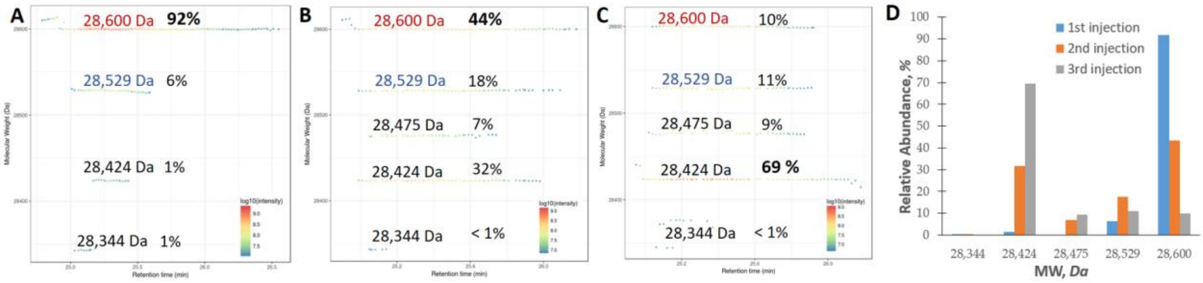

Figure S2. Proteoform map of $\alpha 7$ from human immunoproteasome (i20S) purified from HEK-EBNA cells identified by TD-MS showing the different abundances of each proteoform in three consecutive injections (i.e. with 2-3 hrs difference between each injection (sample was kept at 4°C)). % correspond to the abundance of the proteoform relative to the sum of intensities corresponding to the subunit. (**A**) $\alpha 7$ profile for the first injection showing the main proteoform at 28,600 Da; (**B**) $\alpha 7$ profile for the second injection showing the decrease in abundance of proteoform at 28,600 Da and the increase at 28,424 Da; and (**C**) $\alpha 7$ profile for the third injection showing the main proteoform is now at 28,424 Da. (**D**) A bar plot showing the summary of the abundances of $\alpha 7$ proteoforms from the three consecutive injections.

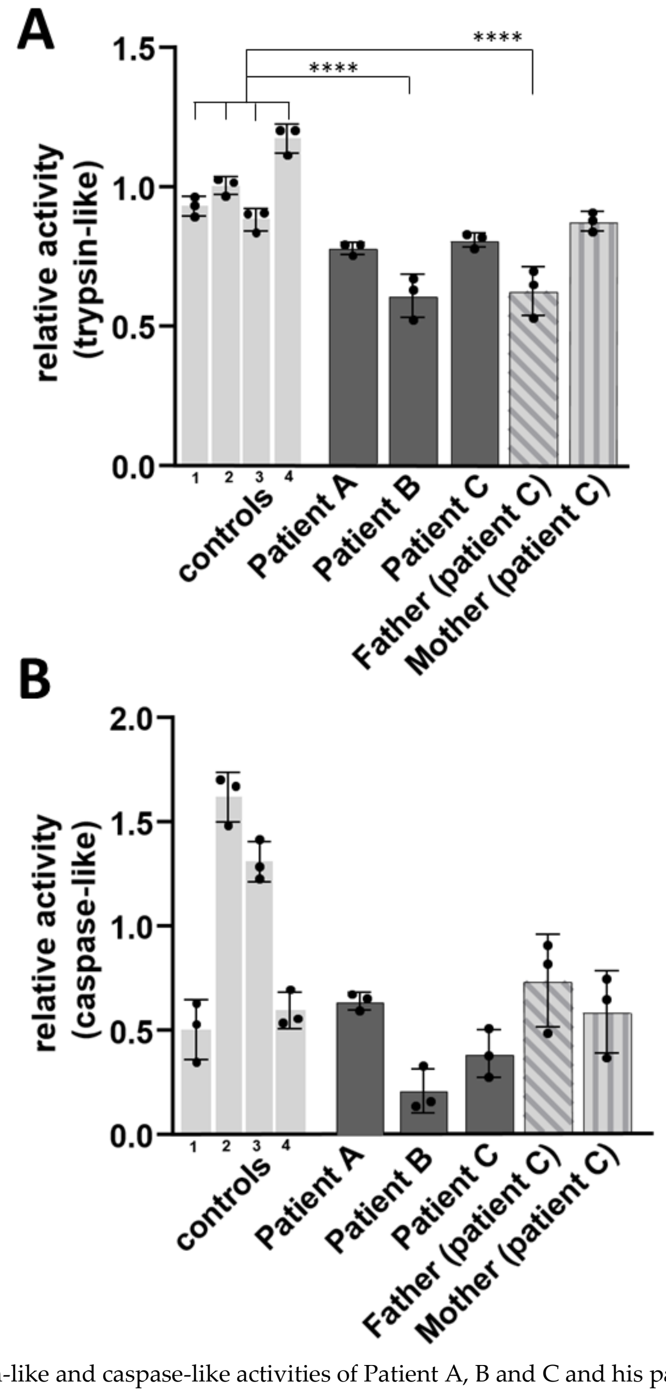

Figure S3. Relative trypsin-like and caspase-like activities of Patient A, B and C and his parents compared to healthy controls: (**A, C, E**) trypsin-like activity using Boc-LRR-AMC substrate; and (B, **D, F**) caspase-like activity using Z-LLE-AMC substrate analyzed in triplicates. (****p-value < 0.0001 from a one-way ANOVA with Tukey HSD test)

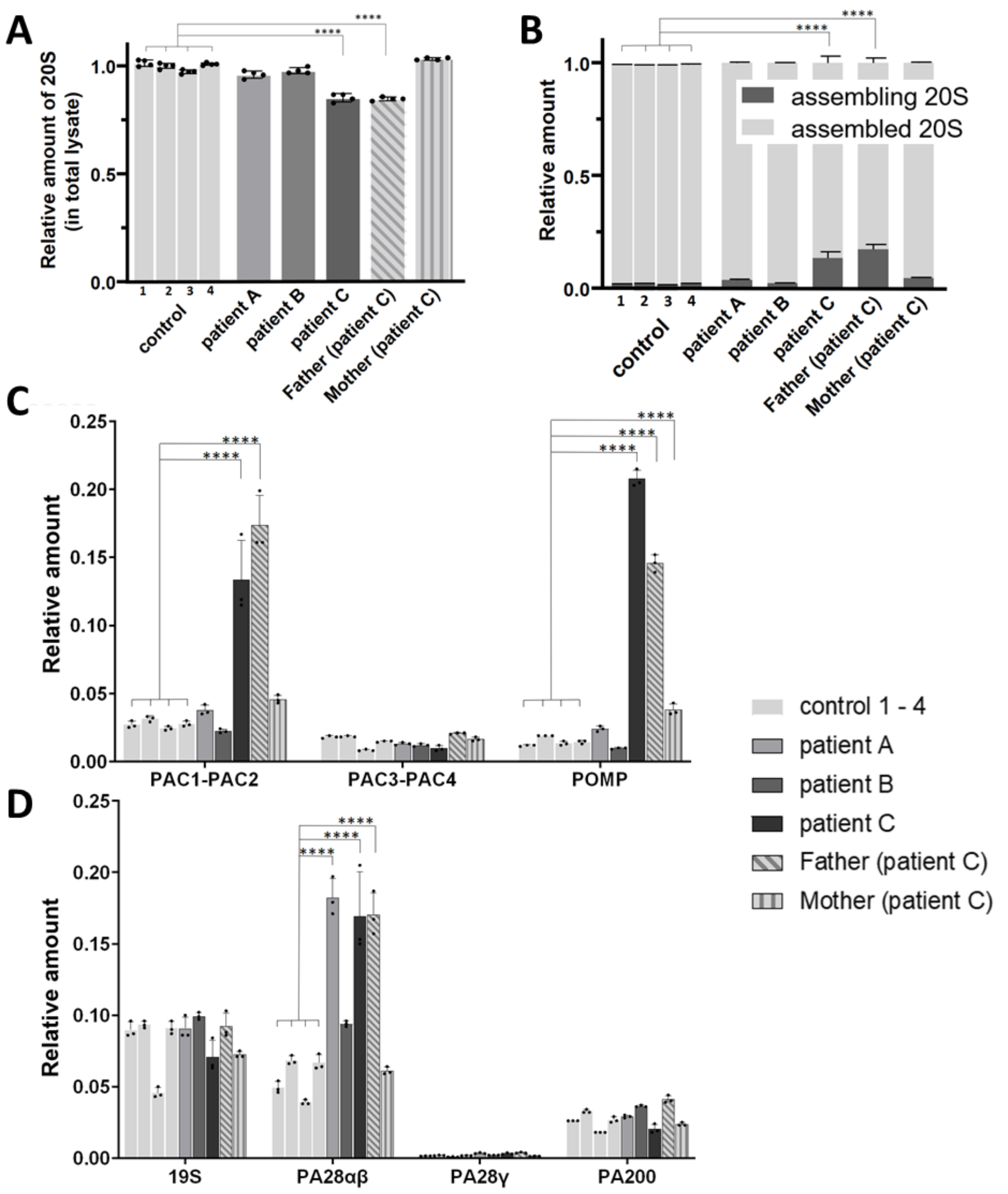

Figure S4. Bottom-up mass spectrometry (BU-MS) analysis of lysates and immunopurified 20S from PRAAS patients: **(A)** Relative amount of 20S of patients compared to healthy controls from total lysate of BLCLs samples analyzed with BU-MS. Dots represent injection replicates; **(B)** Relative amount of assembled and assembling 20S from immunopurified BLCL samples of patients and healthy controls analyzed with BU-MS. The bar represents the average of the injection replicates (3 for the patients and 12 for the controls); **(C)** Amount (relative to the immunopurified 20S non catalytic subunits) of 20S-associated assembly chaperones in immunopurified proteasomes from BLCL samples of patients and healthy controls analyzed with BU-MS. Dots represent injection replicates; and **(D)** The amount (relative to the assembled 20S) of main activators, 19S, PA28$\alpha\beta$, PA28γ and PA200 from immunopurified BLCL samples of patients and healthy controls analyzed with BU-MS. Dots represent injection replicates. (**** p-value < 0.0001 from a one-way ANOVA with Tukey HSD test).

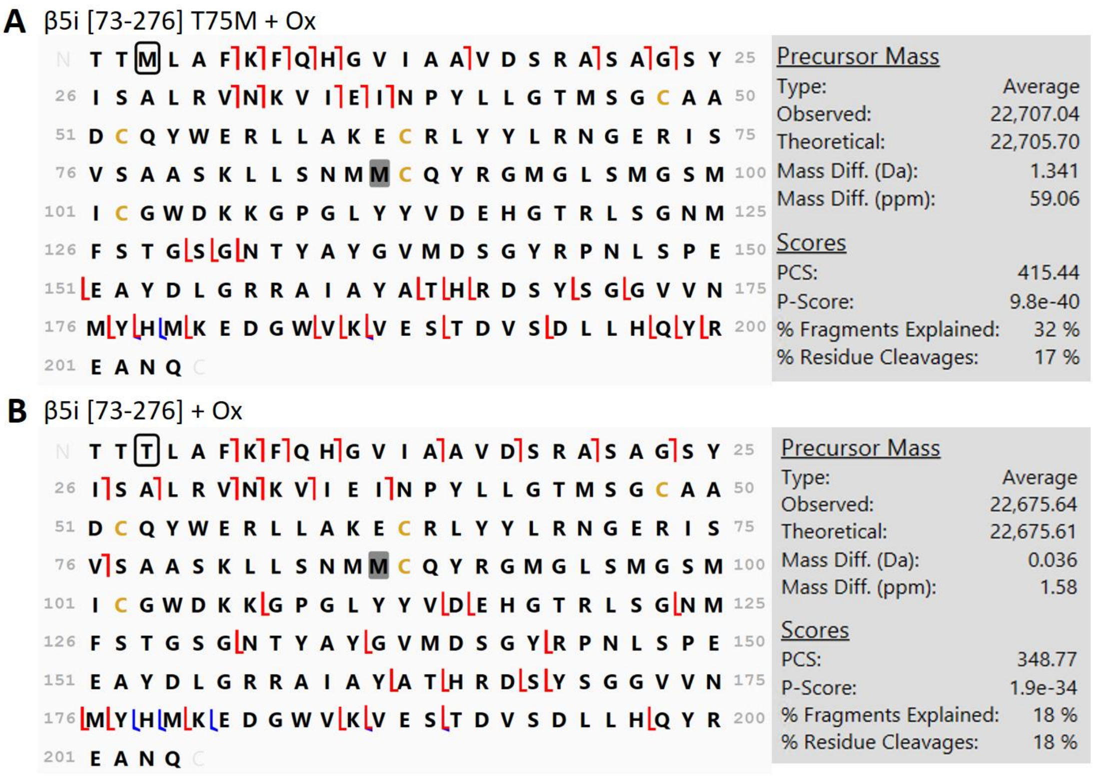

Figure S5. MSMS top-down sequencing of the oxidized forms of (**A**) T75M and (**B**) WT β5i of 20S immunopurified from PRAAS Patient B (β5i T75M and α7 R233del). Note that the oxidation, highlighted in gray, was manually localized on one of the methionines in the middle of the sequence, based on the highest number of c- and z-fragments. The replacement of Thr75 by a Met residue (in box) is clearly confirmed by many *c*- fragments. Red and blue lines correspond to *c*-/*z*- and *b*-/*y*- ions, respectively.

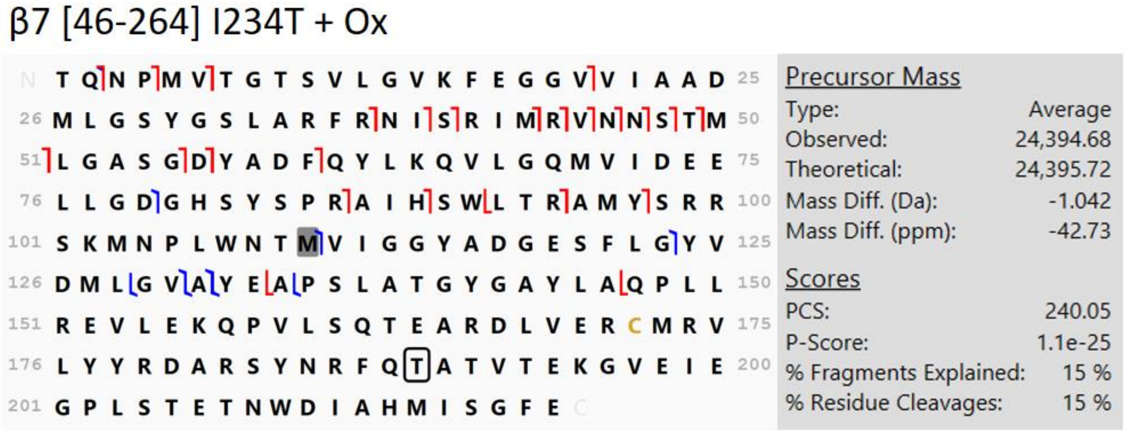

Figure S6. MSMS top-down sequencing of the oxidized form of I234T SNP β7 of 20S from the clinically healthy father (reported to have β7 D212_V214del) of a PRAAS patient with β7 D212_V214del and β7 5'UTR c.-9G>A mutations. Note that the oxidation, highlighted in gray, was manually localized on one of the methionines in the middle of the sequence, based on the highest number of c-/z- and b-/y- fragments. The replacement of I234 by T (in box) in the expressed protein results in a Thr at position 189 in the mature β7 proteoform. Red and blue lines correspond to c-/z- and b-/y- ions, respectively.

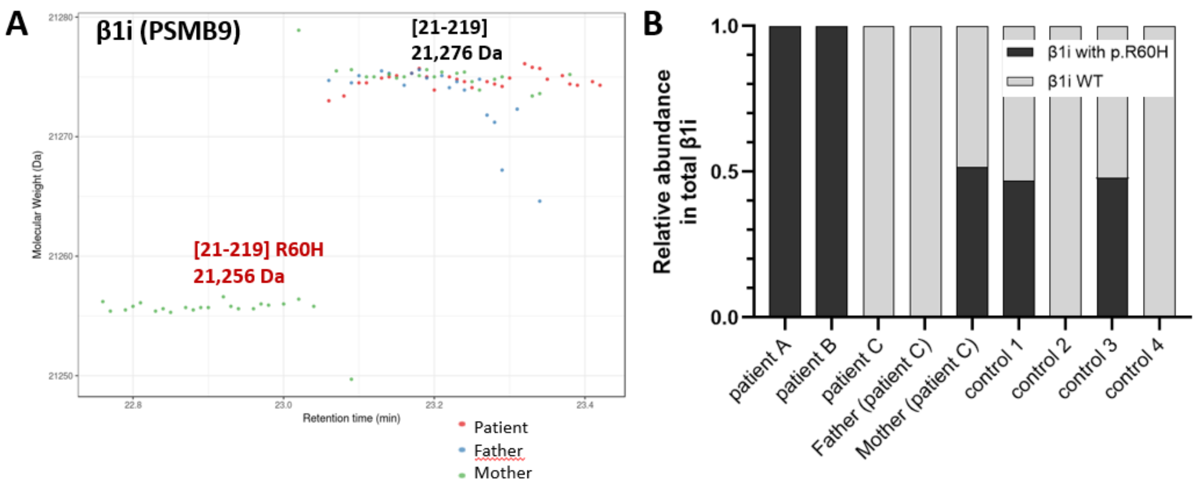

Figure S7. Characterization of β1i (*PSMB9*) proteoforms of immunopurified 20S from PRAAS patients by TD-MS: **(A)** Overlapped proteoform maps of Patient C and his parents, revealing the WT at 21,276 Da and common SNP p.(R60H) at 21,256 Da in the mother. **(B)** Relative abundance of WT β1i and its common SNP p.(R60H).

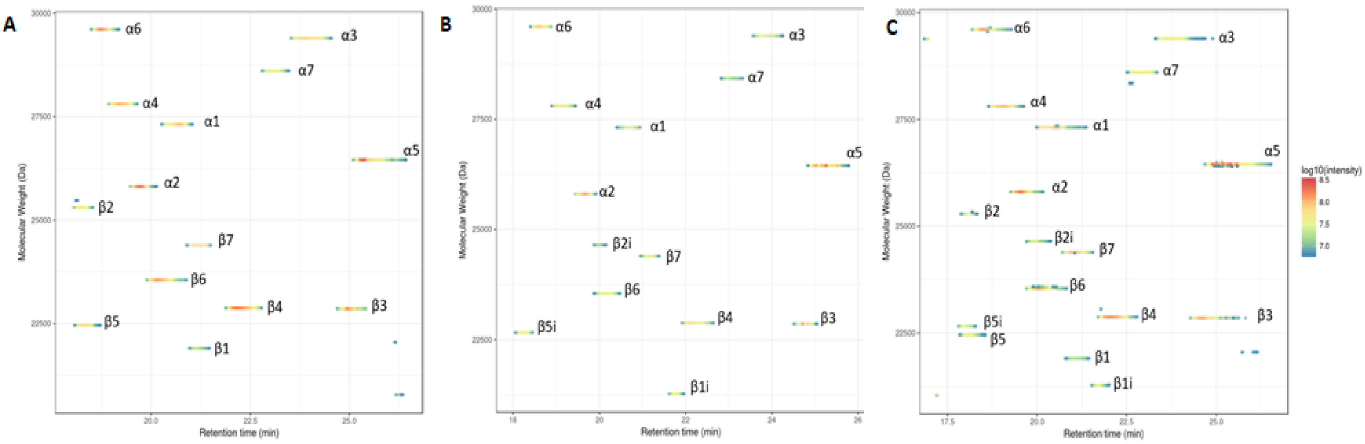

Figure S8. Proteoform maps from VisioprotMS of (**A**) c20S and (**B**) i20S purified from HEK-EBNA cells, and (**C**) 50%:50% c20S:i20S mix analyzed by TD-MS.

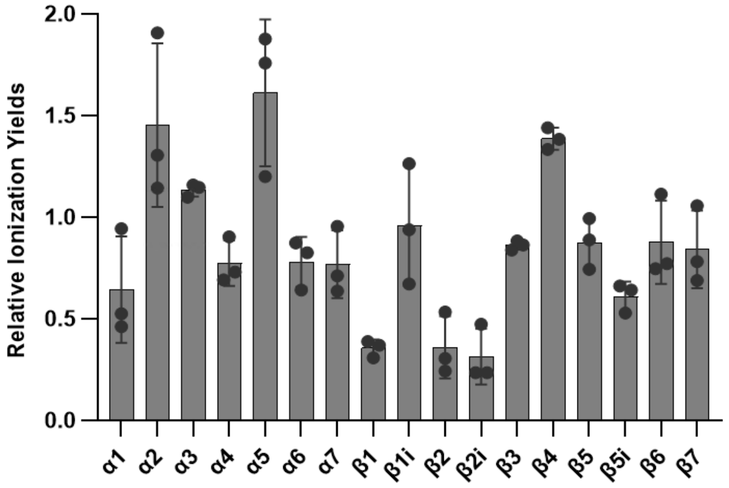

Figure S9. Averaged Relative Ionization Yields (relative to the average intensities of non-catalytic subunits) of each proteasome subunit in TD-MS. Each dot corresponds to the mean of three (catalytic) or six (non-catalytic) measurements from HEK-EBNA cells with either c20S or i20S run in triplicates of injection. Bars and error bars correspond to the mean and standard deviation of three technical replicates run.

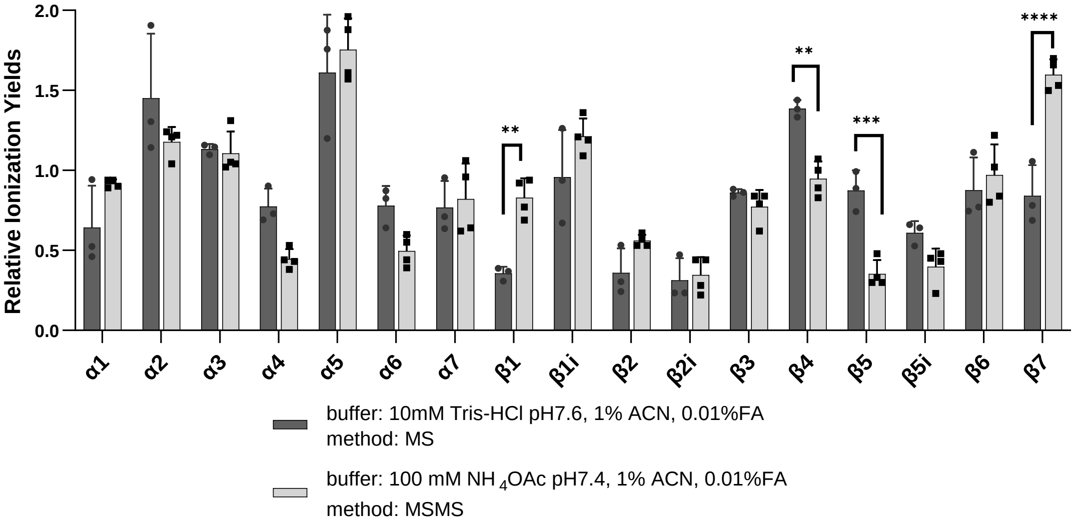

Figure S10. Comparison of relative ionization yields of 20S proteasome subunits in different buffers with different TD-MS acquisition methods ((** p-value ≤ 0.01, *** p-value ≤ 0.001 and ****p-value ≤ 0.0001 from two-way ANOVA with Bonferroni's multiple comparisons test).

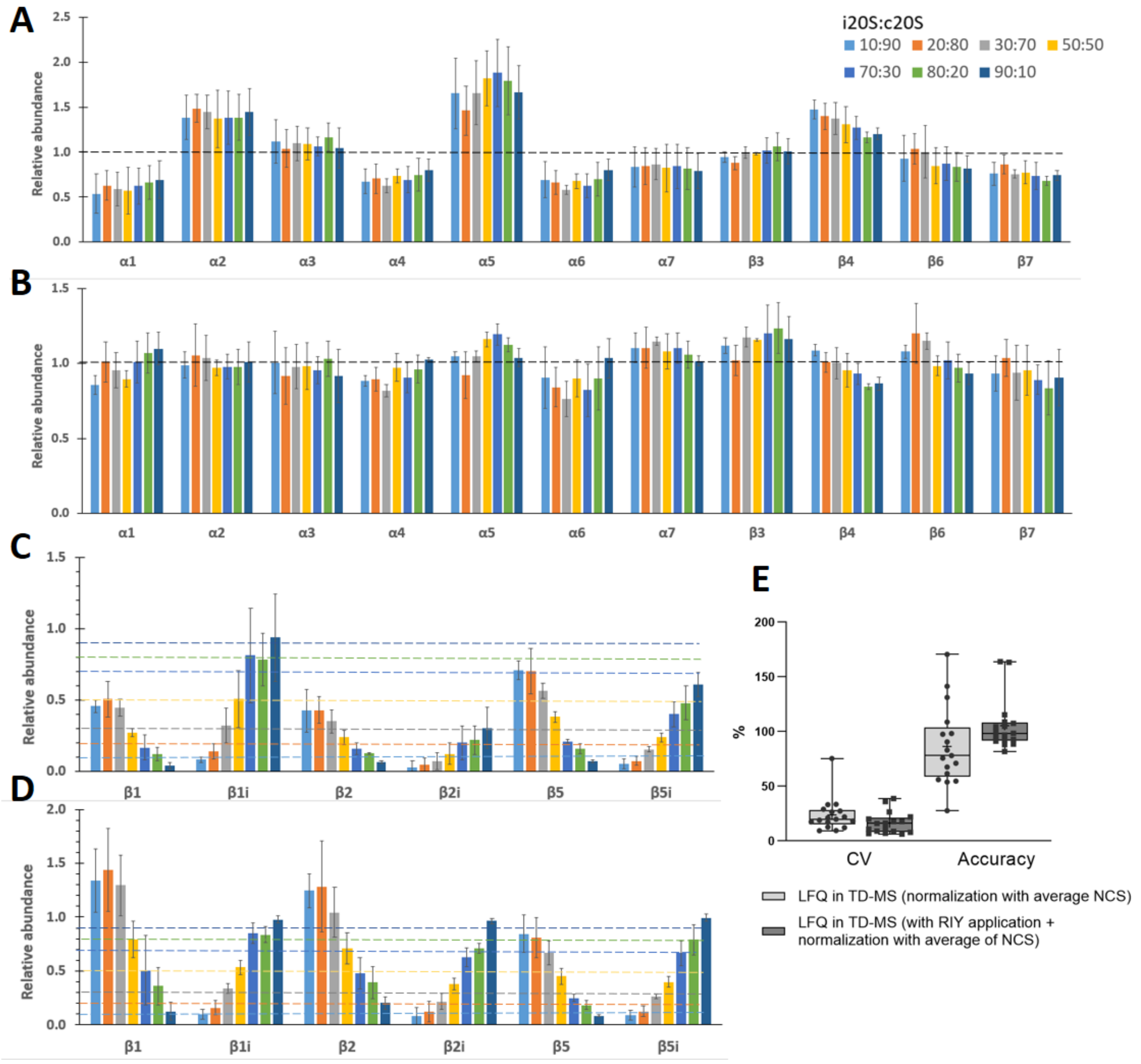

Figure S11. Titration of known c20S and i20S mixes analyzed by TD-MS. Relative amounts calculated for the non-catalytic subunits **(A)** before and **(B)** after the application of RIY. Relative amounts calculated for the catalytic subunits **(C)** before and **(D)** after application of RIY. Error bars are the standard deviations of triplicate measurement. (**E**) Coefficient of variation and accuracy of the amount of proteasome subunits of c20S and i20S mixes calculated by normalizing the intensity with the average of intensities of non-catalytic subunits and by the additional application of the RIY. Each dot in the box plot corresponds to each 20S subunit, the average of three technical replicates performed in triplicates of injection.

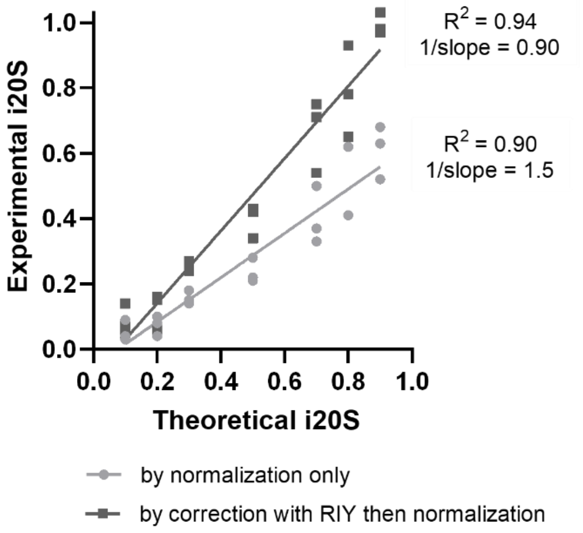

Figure S12. Experimental vs. expected i20S relative abundances obtained by TD-MS with normalization only (light grey) and with RIY correction then normalization (dark grey). The theoretical values are based on our known mixes of c20S and i20S proteasome from HEK EBNA cells.

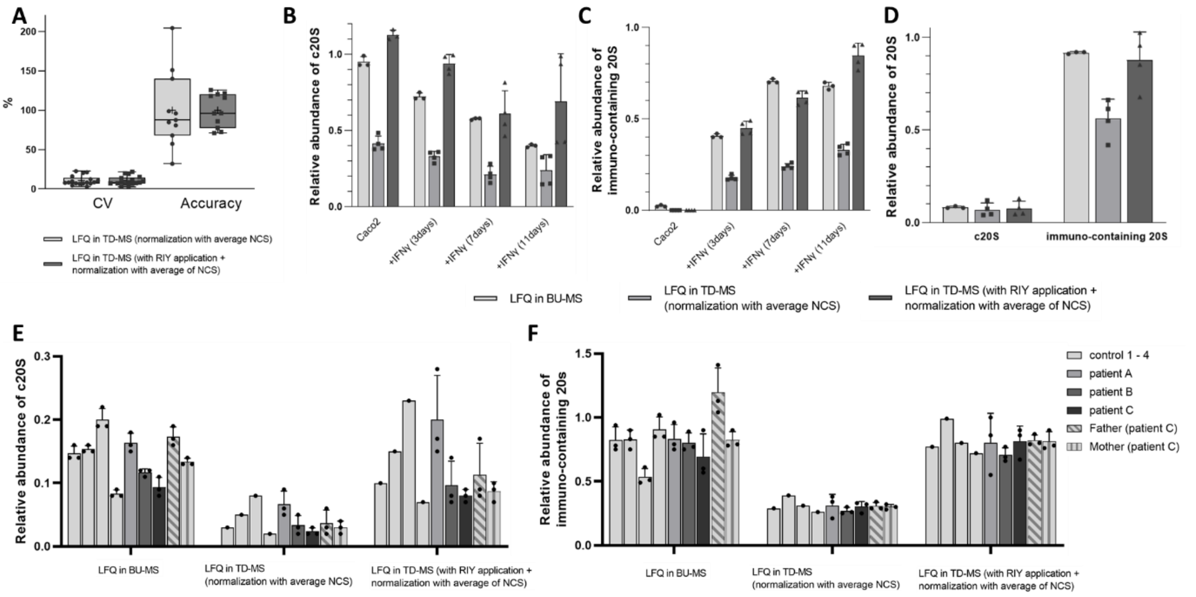

Figure S13. Relative label-free quantification (LFQ) of immunopurified 20S from different samples, showing the benefit of applying the correction of abundances with the relative ionization yield (RIY) in TD-MS. (**A**) Coefficient of variation (CV) and accuracy (non-catalytic subunits or NCS only) of the amount of proteasome in Caco2 cells (± IFNy) calculated by normalizing the intensity with the average of intensities of NCS and by the additional application of the RIY prior to normalization. Each dot in the box plot corresponds to a subunit (average of four technical replicates with three injections each). Relative amount of (**B**) c20S and (**C**) immuno-containing 20S using LFQ from BU-MS and TD-MS (with and without the application of RIY) in Caco2 cells (± IFNy). (**D**) Relative abundance of c20S and immuno-containing 20S in intestinal crypts. Relative amount of (**E**) c20S and (**F**) immuno-containing 20S using LFQ from BU-MS and TD-MS (with and without the application of RIY) in BLCLs derived from PRAAS patients and healthy controls.

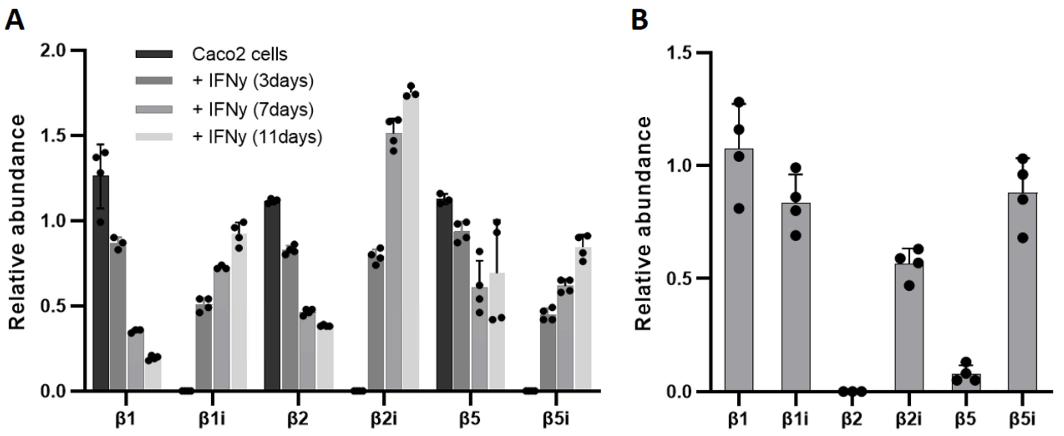

Figure S14. Relative abundances of catalytic subunits obtained with TD-MS in **(A)** IFNy-treated Caco2 cells (after 3, 7, and 11 days of treatment) and **(B)** intestinal crypts, obtained by correcting with the RIY then normalizing with the average abundance of non-catalytic subunits.

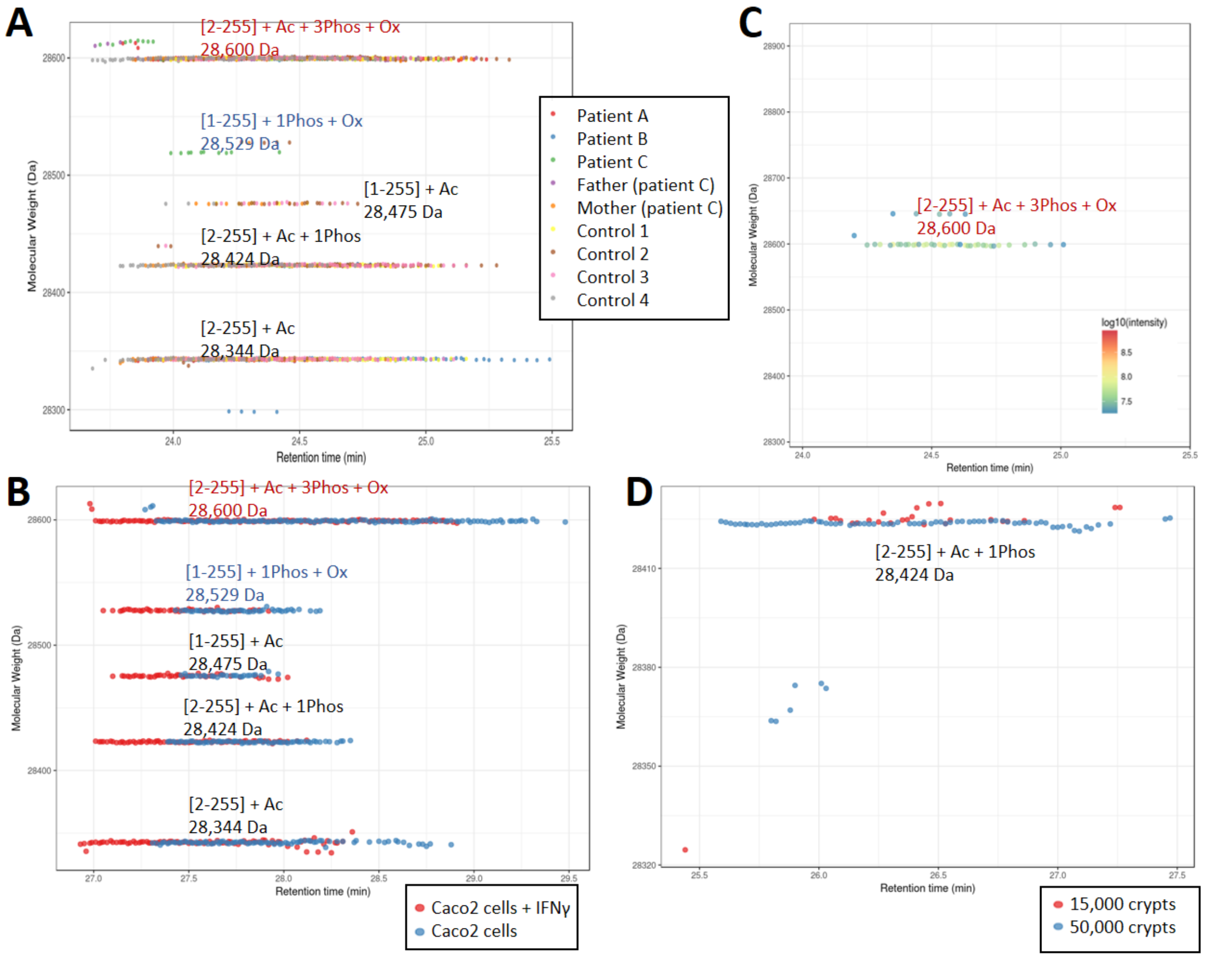

Figure S15: The highly unstable triply phosphorylated α7 proteoform is found by TD-MS in samples containing both c20S and i20S including **(A)** BLCL derived from PRAAS patients and controls, **(B)** Caco2 cells ± IFNy, **(C)** lung organoïds, but not from **(D)** intestinal crypts, which were stored for several months at -80°C.
